# *In Vitro *Antibacterial and Time-Kill Assessment of Crude Methanolic Stem Bark Extract of *Acacia mearnsii *De Wild against Bacteria in Shigellosis

**DOI:** 10.3390/molecules17022103

**Published:** 2012-02-21

**Authors:** Olufunmiso Olusola Olajuyigbe, Anthony Jide Afolayan

**Affiliations:** Phytomedicine Research Centre, Department of Botany, University of Fort Hare, Alice, 5700, South Africa; Email: funmijuyigbe12@yahoo.com

**Keywords:** *A. mearnsii*, antibacterial, shigellosis, time-kill, methanolic extract

## Abstract

Shigellosis is an important cause of worldwide morbidity and mortality among young children and old people for which treatment with antimicrobial agents is limited. Hence, the need for curative potentials obtainable from medicinal plants becomes inevitable. This study was carried out to assess the antibacterial potentials of crude methanolic extract of the stem bark of *Acacia mearnsii* against some selected bacteria of clinical importance in shigellosis. The bacteria were inhibited by the extract to produce concentration dependent inhibition zones. The extract exhibited a varied degree of antibacterial activity against all the tested isolates. The MIC values for Gram negative (0.0391–0.3125) mg/mL and those of Gram positive bacteria (0.0781–0.625) mg/mL indicated that the Gram negative bacteria were more inhibited by the extract than the Gram positive bacteria. Average log reduction in viable cell count in time-kill assay ranged between −2.456 Log_10_ to 2.230 Log_10_ cfu/mL after 4 h of interaction, and between −2.921 Log_10_ and 1.447 Log_10_ cfu/mL after 8 h interaction in 1× MIC and 2× MIC of the extract. The study provided scientific justification for the use of the crude methanolic extract from the stem bark of *A. mearnsii* in shigellosis. The degree of the antibacterial activity indicated that the crude extract is a potential source of bioactive compounds that could be useful for the development of new antimicrobial agents capable of decreasing the burden of drug resistance and cost of management of diseases of clinical and public health importance in South Africa.

## 1. Introduction

Shigellosis, an important cause of worldwide morbidity and mortality [1], is a disease of public health importance in developing countries causing self-limited diarrhea to severe dysentery [2]. It is primarily a disease of poor, crowded communities that do not have adequate sanitation or clean water [3]. Aside from clinical intestinal manifestations, it causes a wide variety of extra-intestinal signs such as bacteremia or neurologic manifestations [4]. Though Enteroinvasive *Escherichia coli *(EIEC) and Enterohemorrhagic *E. coli *(EHEC) are less frequently implicated in bacillary dysentery [5], Enteropathogenic bacteria are mainly implicated [6]. Barman *et al.* [7] and Vinh *et al.* [8] indicated, also, that shigellosis is caused by different species of Shigella (*Shigella flexneri, Shigella dysenteriae, Shigella boydii* and* Shigella sonnei*) belonging to the family Enterobacteriaceae. *Shigella sonnei* is the serogroup of Shigella most frequently responsible for sporadic and epidemic enteritis in developed countries while *S. flexneri* is the most dominant strain in developing countries [9]. EHEC known as verocytotoxin producing *E. coli* (VTEC) [10] produce a Shiga-like toxin (SLT) and the enteroinvasive *E. coli *(EIEC) possess genetic and biochemical characteristics similar to *Shigella* [11]. Although shigellosis is a major source of gastroenteritis throughout the world with *Shigella spp.* being majorly implicated [12], other gastrointestinal pathogens such as Salmonella, Yersinia, enteropathogenic *Escherichia coli *(EPEC), *Entamoeba hystolytica*, *Bacillus subtilits*, *Bacillus cereus*, *Aeromonas hydrophyla* and *Campylobacter sp.* have been involved.

Epidemiologically, 165 million children and young adults suffer worldwide annually with 1 million associated deaths from shigellosis. 99% of these cases occur in developing countries [13,14] with 69% occurring in children aged less than five years [15,16]. While Lee *et al.*, [17], Niyogi, [14] and Wang *et al.*, [18,19] reported that there is still a relatively high incidence of bacillary dysentery among children and old people, Alam and Bhatnagar, [20], Seol *et al.*, [21] and Von Seidlein* et al.*, [22] indicated that easy variability of the pathogen, lack of cross-immunity among different types of Shigella bacteria, increase of drug-resistant strains among the carriers and the patients’ immunity status that cannot be sustained after the infection are responsible for the observed incidence rate among the young children and old people.

*Shigella spp.* and most of the associated gastrointestinal pathogens are usually spread directly from person to person by the fecal–oral route or indirectly by ingestion of fecal contaminated food or water [23,24]. The pathogenesis showed that an inoculum of 10^2^ *Shigella *cells and as many as 10^6^ EIEC cells are sufficient for the onset of the infection [25]. Though both EIEC and *Shigella *cause bacillary dysentery in hu­mans by invading and multiplying within epithelial cells of the colonic mucosa to cause an intense inflamma­tory response characterized by abscesses and ulcerations [26,27], early detection and intervention in disease outbreaks has enabled timely public health measures to limit illness and death [28]. This could be in addition to improved nutrition in many countries, improved healthcare delivery in some areas and more widespread use of measles vaccine [29], vitamin A supplementation [30] and proper case management [31] to reduce the severity of intestinal infections.

Though shigellosis is a global human health problem and a public health challenge, especially in endemic areas, emergence of multi-drug resistant (MDR) strains involving all microbial pathogens and antimicrobial drugs is a simultaneous growing concern globally [2] and multidrug resistance in shigellosis is not exempted [14]. Due to the global emergence of drug resistance, the choice of antimicrobial agents for treating shigellosis is limited. Hence, the curative potentials of medicinal plants locked-up and embedded in some chemical components that effect physiological responses are needed for the treatment of the infection.

Consequently, in the last few decades, the study of medicinal plants and their traditional use in different parts of the World has increased [32]. Hundreds of plants have been used as herbal remedies in indigenous medicine systems [33]. While herbal medicines are assumed to be of great importance in the primary healthcare of individuals in rural communities [34,35], plant-based traditional knowledge coupled with the high cost involved in the development of patentable chemicals and drugs [36] are recognized as essential tools in search for new sources of drugs and neutraceuticals [37]. Thus, antimicrobial activity of crude and semi-purified extracts of many plants has been widely reported [38,39,40,41]. The increasing use of traditional therapies which the laypeople considered as a part of their heritage [42] now requires more scientifically sound evidence for the principles behind plants’ therapeutic effectiveness in complementary and alternative medicines [43].

The genus *Acacia* (Wattle) is a member of the pea family (Leguminosae). While it is the largest group of vascular plants in Australia with about 1,000 species currently recognized, it is a group of 1,404 species distributed throughout tropical and warm temperate areas of the World. Leaves, twigs and bark are used for a range of medicinal purposes, including decoctions for the treatment of flu, colds, skin ailments and smoke from certain species has been used to promote health. Seeds of about 30 species are used as food [44], and many acacias are used as stock fodder in the arid zone rangelands [45,46]. Though *Acacia* with diverse morphological, biological and ecological attributes offers great scope for economic, environmental and social utilization, *Acacia mearnsii* is basically known as one of the World’s highest yielding sources of high quality condensed tannin. Its pharmacological importance is less explored despite its being grown in large-scale plantations in South Africa and other parts of the world. This study was designed to assess its antibacterial activity and justify its relevance in the treatment of dysentery for which it has been implicated in the rural areas in South Africa.

## 2. Results and Discussion

**Figure 1:**
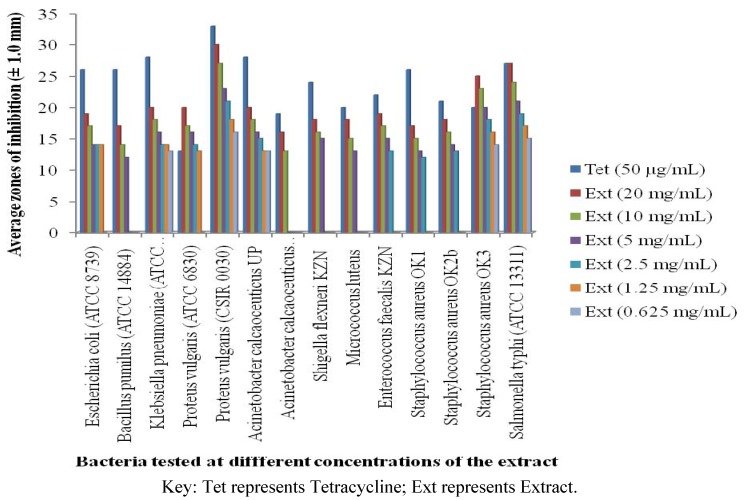
Bacterial susceptibility to different concentrations of crude methanolic extract of *A. mearnsii*.

The antibacterial activity of the stem bark methanol extract of *A. mearnsii* was evaluated by measuring the inhibition zone against medically important pathogens. The degree of the antibacterial activity was assayed by the serial two fold dilution method to determine the minimum inhibitory concentration (MIC) of the extract while the bactericidal activity was assessed by time kill assay *in vitro*. The result showed that the extract had good antibacterial activity against both Gram positive and Gram negative bacteria at different concentrations (Figure 1). The agar diffusion assay indicated that the bacteria were inhibited by the extract to produce concentration dependent inhibition zones. At the least used concentration, five of the bacteria were inhibited. At the highest concentration, all the bacteria were inhibited. The inhibition zones produced by 100 µL of 20 mg/mL of the extract ranged between 16 mm and 30 mm for all the bacteria. Of the Gram negative bacteria, *Salmonella typhi* (ATCC13311) and *Proteus vulgaris* (CSIR 0030) had the highest inhibition zones of 27 mm and 30 mm, respectively, while those of other Gram negative bacteria ranged between 16 mm and 20 mm. Of the Gram positive bacteria, *Staphylococcus aureus* OK_3_ had the highest inhibition zone (25 mm) while those of others ranged between 17 mm and 18 mm. The extract exhibited a varied degree of antibacterial activity against all the tested isolates for which the MIC values ranged between 0.0391 mg/mL and 0.625 mg/mL. While the MIC values for Gram negative ranged between 0.0391 mg/mL and 0.3125 mg/mL, those of Gram positive bacteria ranged between 0.0781 mg/mL and 0.625 mg/mL. The MBC values were similar or 2–4 folds higher than MIC values for the extract (Table 1). While a good agreement exists between the agar diffusion and macrobroth dilution assays, the antibacterial effects showed that Gram negative bacteria were more inhibited by the extract than the Gram positive bacteria. The extract was less potent than the standard antibiotic, tetracycline, of which 100 µL of 50 µg/mL had produced inhibition zones ranging between 13 mm and 33 mm in diameter. The variation between the activities of the extract and the standard antimicrobial drug may be due to the mixtures of bioactive compounds present in the extract compared to the pure compound contained in the standard antibiotics that rarely have the same degree of activity as the unrefined extract at comparable concentrations or dose of the active component.

The results of time-kill studies are presented in Table 2. Data are presented in terms of the log_10_ cfu/mL change and are based on the conventional bactericidal activity standard, that is, a 3 Log_10_ cfu/mL or greater reduction in the viable colony count [47]. Average log reduction in viable cell count in time-kill assay ranged between −2.456 Log_10_ to 2.230 Log_10_ cfu/mL after 4 h of interaction, and between −2.921 Log_10_ and 1.447 Log_10_ cfu/mL after 8 h interaction in 1× MIC and 2× MIC of the extract. The greatest reduction in cell density with the extract was observed with *Klebsiella pneumoniae * (10031) (−2.921 log_10_), *Acinetobacter calcaoceuticus* UP (−2.638 Log_10_), *Micrococcus luteus* (−2.444 Log_10_), *Acinetobacter calcaoceuticus* CSIR (2.387Log_10_) and *Enterococcus faecalis* KZN (−2.246 Log_10_). At these concentrations, the significant reduction in the bacterial population suggests that the extract was highly bactericidal after a 4 h incubation period, while the bacterial colonies were almost wiped out after incubating for 8 h. On the contrary, there was a net growth of all the test isolates when subjected to ½ MIC concentration of the extract. Growth inhibition and efficacy of the crude methanolic stem bark extract were observed to be dose and time dependent producing distinct time-kill profiles for the tested bacteria.

In the last few decades, the acceptance of traditional medicine as an alternative form of health care and the development of microbial resistance to the classical antibiotics have led researchers to investigate the antimicrobial activity of several medicinal plants utilized as popular folk medicines or finished products collectively known as phytomedicines. While the continuous spread of multidrug-resistant pathogens has become a serious threat to public health and a major concern for infection control practitioners worldwide [48], re-emergence of previously controlled diseases contributes substantially to the high frequency of opportunistic and chronic infection cases in developing countries [49,50]. Hence, since resistance to currently available antimicrobial agents used to treat infections requires new appraisals [51,52], *in vitro* susceptibility testing and determination of the bactericidal activity with the aim of indicating the efficacy of an antimicrobial agent against infecting organisms to provide informed decision regarding treatment of serious infections become necessary. The reason being that, in certain unique clinical settings involving sites not easily accessed by host defenses [53,54] and neutropenic immunosuppressed hosts [55], the ability of the agent administered to kill the pathogen outright may be quite important [56,57].

**Table 1:** Antibacterial activity of the methanolic extract of *Acacia mearnsii *De Wild.

	Tetracycline (µg/mL)	Methanolic extract (mg/mL)		
	MIC	MIC	MBC	MIC/MBC
*Escherichia coli* (ATCC 8739)	0.977	0.3125	0.625	2
*Bacillus pumilus* (ATCC 14884)	3.125	0.3125	0.625	2
*Klebsiella pneumoniae* (ATCC 10031)	0.488	0.1563	0.1563	1
*Proteus vulgaris* (ATCC 6830)	7.8125	0.1563	0.625	4
*Proteus vulgaris* (CSIR 0030)	0.488	0.0391	0.1563	2
*Acinetobacter calcaoceuticus* UP	125	0.1562	0.3125	2
*Acinetobacter calcaoceuticus anitratis* CSIR	0.0305	0.3125	0.625	2
*Shigella flexneri* KZN	0.1966	0.0391	0.0781	2
*Micrococcus luteus*	31.25	0.078	0.1563	2
*Enterococcus faecalis* KZN	15.625	0.156	0.3125	2
*Staphylococcus aureus* OK_1_	0.976	0.1563	0.625	4
*Staphylococcus aureus* OK2_b_	0.1966	0.625	1.25	2
*Staphylococcus aureus* OK_3_	0.1953	0.3125	0.625	2
*Salmonella typhi* (ATCC 13311)	0.0305	0.3125	0.3125	1

**Table 2:** *In vitro* time kill assessment of the rude methanolic stem bark of *A. mearnsii*.

	Log_10_Kill			Log_10_Kill			Log_10_Kill		
	½ X MIC			MIC			2 X MIC		
	0 h	4 h	8 h	0 h	4 h	8 h	0 h	4 h	8 h
*Escherichia coli* (ATCC 8739)	2.083	3.131	4.248	2.199	1.294	1.125	2.340	0.121	−1.542
*Bacillus pumilus* (ATCC 14884)	2.212	3.422	4.449	2.392	2.167	1.033	2.422	0.111	−1.815
*Klebsiella pneumoniae* (ATCC 10031)	2.170	3.386	4.292	2.207	1.121	0.427	2.185	−1.456	−2.921
*Proteus vulgaris* (ATCC 6830)	2.164	3.401	4.468	2.217	1.187	0.274	2.200	1.132	−1.951
*Proteus vulgaris* (CSIR 0030)	ND	ND	ND	ND	ND	ND	ND	ND	ND
*Acinetobacter calcaoceuticus* UP	2.091	2.237	3.427	2.375	1.185	0.233	2.345	−1.0915	−2.638
*Acinetobacter calcaoceuticus anitratis* CSIR	2.276	3.324	4.519	2.354	1.199	0.122	2.375	−0.951	−2.387
*Shigella flexneri* KZN	ND	ND	ND	ND	ND	ND	ND	ND	ND
*Micrococcus luteus*	2.193	2.093	3.137	2.241	1.420	0.121	2.210	0.292	−2.444
*Enterococcus faecalis* KZN	2.272	2.121	3.179	2.272	1.358	0.246	2.265	0.130	−2.246
*Staphylococcus aureus* OK_1_	2.386	2.203	3.322	2.268	2.140	1.032	2.321	1.083	−1.614
*Staphylococcus aureus* OK2_b_	2.234	2.025	3.340	2.336	1.137	0.053	2.303	1.358	−1.287
*Staphylococcus aureus* OK_3_	3.342	4.690	6.633	3.167	2.230	1.447	3.248	1.916	1.062
*Salmonella typhi* (ATCC 13311)	2.292	2.328	3.155	2.270	2.201	1.274	2.288	2.185	−0.727

Key: ND represents Not Determined.

In this study, our data showed that the response of the bacteria to the tested extract varied among the strains and are concentration and time dependent. The differences in susceptibility may be due to the differences in cell wall composition and/or genetic content of their plasmids [58]. While the active components in the crude extract may be acting synergistically to produce good antimicrobial effects [59], the disparity between the activities of the extract and the standard antimicrobial drug may be due to the mixtures of bioactive compounds present in the extract compared to the pure compound contained in the standard antibiotics [60]. The MBC values obtained suggested that a biocidal effect of the crude extract could be expected on most of the tested organisms [61,62]. This effect which could necessitate isolation and development of new antimicrobial agents was exhibited by the time-kill assessment of the extract. The observed degree of antibacterial activity may be attributed to the inherent astringent property associated with the high percentage of tannin sometimes called polyphenols [63,64] in the plant while its phytochemical assessment showed that both total phenolic content and the antioxidant activity correlated well [65]. Thus, in agreement with other reports, the antibacterial activity of the extract may be due to the presence of phytoconstituents such as tannin [66], alkaloids [67], terpenoids [68], flavonoids [69,70], and saponins [71,72].

In addition, the differences between the susceptibility of the Gram positive and Gram negative bacteria may be attributed to the differences in their cell wall components and thicknesses [73]. However, the fact that Gram negative bacteria were more susceptible to the extract is significant as Gram positive bacteria are usually reported as being more affected by plant extracts [74,75,76]. In the Gram negative bacteria, the extract was able to overcome the permeability barrier provided by the cell wall and the membrane accumulated resistance mechanisms [77]. Though several mechanisms of action in the growth inhibition of bacteria including destabilization of cytoplasmic and plasma membranes, inhibition of extracellular microbial enzymes and metabolisms, and deprivation of the substrate required for microbial growth [78] have been reported, the mode of antimicrobial action of plant tannin may be related to their ability to inactivate microbial adhesions, enzymes, cell envelope transport proteins, and mineral uptake [79] or polymerization through oxidation reactions [80]. Hence, the disintegrative ability of tannin compounds on the bacterial colonies probably resulted from their interference with the bacterial cell wall to inhibit their growth [81,82] while the degree of toxicity resulting from their polymerization may be responsible for the degree of the bactericidal activity of the extract over a short period of time in time-kill assay.

## 3. Experimental

### 3.1. Collection of Plant Material

The bark materials of *Acacia mearnsii *De Wild were collected in August, 2010, from the plant growing within the University of Fort Hare campus in Alice, South Africa. The plant was authenticated in the Department of Botany and a voucher specimen (OLAJ Med 2010/01) was prepared and deposited in the Griffen Herbarium of the University.

### 3.2. Extract Preparation

The bark sample was air-dried at room temperature and pulverized using a milling machine. The extract of the bark material was prepared in accordance to the description of Basri and Fan [83]. About 100 g of the pulverized sample was extracted with 500 mL of methanol for 48 h with shaking (Stuart Scientific Orbital Shaker, Staffordshire, UK). The extract was filtered through Whatman No. 1 filter paper and concentrated under reduced pressure at 40 °C using a rotary evaporator (Laborota 4000–efficient, Heldolph, Germany). The crude extract collected was allowed to dry at room temperature to a constant weight of 18.7 g. The extract was redissolved in dimethylsulfoxide (DMSO) to the required concentrations for bioassay analysis. The reconstituted extract solution was sterilized by filtering through 0.45 μm membrane filter and tested for sterility after membrane filtration by introducing 2 mL of the extract into 10 mL of sterile nutrient broth before being incubated at 37 °C for 24 h. A sterile extract was indicated by the absence of turbidity in the broth after the incubation period.

### 3.3. Test Organisms and Bacterial Inocula Preparation

The bacteria used in this study included *Escherichia coli* (ATCC 8739), *Klebsiella pneumoniae* (ATCC 10031), *Bacillus pumilus* (ATCC 14884), *Proteus vulgaris* (ATCC 6830), *Proteus vulgaris* (CSIR 0030), *Acinetobacter calcaoceuticus* UP, *Acinetobacter calcaoceuticus anitratis* CSIR, *Shigella flexneri* KZN, *Salmonella typhi* (ATCC 13311), *Micrococcus luteus, Enterobacter faecalis* KZN, *Staphylococcus aureus* OK_2b_, *Staphylococcus aureus* OK_1_ and *Staphylococcus aureus* OK_3_. These strains were obtained from the Department of Biochemistry and Microbiology, University of Fort Hare, Alice, South Africa. The inocula of the test bacteria were prepared using the colony suspension method [84]. Colonies picked from 24 hold cultures grown on nutrient agar were used to make suspensions of the test organisms in saline solution to give an optical density of approximately 0.1 at 600 nm. The suspension was then diluted 1:100 by transferring 0.1 mL of the bacterial suspension to 9.9 mL of sterile nutrient broth before being used.

### 3.4. Antimicrobial Assay by Agar Diffusion Method (Inhibition Zones)

For the initial determination of the antibacterial activity of the crude methanolic extract of *A. mearnsii*, the susceptibility screening of the test bacteria to the extract and tetracycline, used as control, was determined using the modified Kirby-Bauer diffusion technique [85] by swabbing Mueller-Hinton agar (MHA) (Oxoids Ltd, Basingstoke, Hampshire, UK) plates with the resultant saline suspension of each adjusted bacterial strain. Wells were then bored into the agar media using a sterile 6 mm cork borer. The wells were filled with 100 µL of different concentrations of the extract and tetracycline taking care not to allow spillage of the solutions onto the surface of the agar. The culture plates were allowed to stand on the laboratory bench for 1 h to allow proper diffusion of these solutions before being incubated at 37 °C for 24 h. Wells in blank Mueller Hinton agar containing 10% DMSO representing the final concentration of the DMSO in the test plates without the extract served as positive controls. The determinations were done in duplicates. After 24 h of incubation, the plates were examined if there is any inhibition zone [86]. The organisms were not susceptible to 10% DMSO used in the control assay. The diameters of the inhibition zones produced by each of the concentrations of the solutions were measured in millimeters [87] and interpreted using the CLSI zone diameter interpretative standards [88].

### 3.5. Macrobroth Dilution for Minimum Inhibitory Concentration (MIC)

Minimum inhibitory concentration (MIC) defined as the lowest concentration which resulted in maintenance or reduction of inoculums viability [89] was determined by serial tube dilution technique [90,91] for the bacterial isolates. Different concentrations (0.02–10.0) mg/mL of the extract and (0.03–250) µg/mL of tetracycline were differently prepared by serial dilutions in Mueller Hinton broth medium. Each tube was then inoculated with 100 µL of each of the adjusted bacterial strain. Two blank Mueller Hinton broth tubes, with and without bacterial inoculation, were used as the growth and sterility controls. The bacterial containing tubes were incubated aerobically at 37 °C for 24 h. After the incubation period, the tubes were observed for the MICs by checking the concentration of the first tube in the series (ascending extract and antibiotic concentrations) that showed no visible trace of growth. The first tube in the series with no visible growth after the incubation period was taken as the MIC. 

### 3.6. Determination of Minimum Bactericidal Concentrations (MBC)

Since the clinical occurrences of tolerance usually necessitate bactericidal testing, the MBC was determined by sampling all the macroscopically clear tubes and the first turbid tube in the series. Before being sampled, the tubes were gently mixed by flushing them with a sterile pipette, and a 100 μL aliquot was removed. Each aliquot was placed on a single antibiotic-free nutrient agar plate in a single streak down the center of the plate in accordance with the method of Shanholtzer *et al.*, [92]. The samples were allowed to be absorbed into the agar until the plate surface appeared dry (about 30 min). The aliquot was then spread over the plate by lawning technique. In many studies on microbial susceptibility, this subculturing method has been found satisfactory in eliminating the problem of antimicrobial agent carryover from the 100 μL subculture volume [93,94,95,96]. The growth and sterility controls were sampled in the same manner. The MBC lawned plates were incubated for 24 h at 35 °C. After the incubation periods, the lowest concentrations of the extract that did not produce any bacterial growth on the solid medium were regarded as MBC values for this extract [97]. This observation was matched with the MIC test tube that did not show evidence of growth after 48 h of incubation.

### 3.7. Determination of Rate of Kill

Assays for the rate of killing bacteria by the crude methanolic extract were carried out using a modified plating technique of Eliopoulos amd Eliopoulos [98] and Eliopoulos and Moellering [99]. The extract was incorporated into 10 mL Mueller Hinton broth in McCartney bottles at ½ MIC, MIC and 2× MIC. Two controls, one Mueller Hinton broth without extract inoculated with test organisms and Mueller Hinton broth incorporated with the extract at the test concentrations without the test organisms, were included. Inoculums density, approximately 10^5^ cfu/mL further verified by total viable count, was used to inoculate 10 mL volumes of both test and control bottles. The bottles were incubated at 37 °C on an orbital shaker at 120 rpm. A 100 µL aliquot was removed from the culture medium at 0, 4 and 8 h for the determination of cfu/mL by the plate count technique [100] by plating out 25 µL of each of the dilutions. The problem of extract carryover was addressed by dilution as described previously by Pankuch *et al.*, [101]. After incubating at 37 °C for 24 h, emergent bacterial colonies were counted, cfu/mL calculated and compared with the count of the culture control without extract.

## 4. Conclusions

The results of the present study provided scientific justification for the use of the methanol extract from the stem bark of *Acacia mearnsii* in shigellosis. The degree of the antibacterial activity indicated that the crude extract is a potential source of bioactive compounds that could be useful for the development of new antimicrobial agents capable of decreasing the burden of drug resistance and cost of management of diseases of clinical and public health importance in this South Africa. Since plants produce a diverse range of bioactive molecules making them rich sources of different types of medicines, further pharmacological and toxicity will be necessary to establish their safety as antimicrobial agents.
